# (*m*-Phenyl­enedimethyl­ene)bis­(triphenyl­phospho­nium) bis­[chlorido(penta­fluoro­phen­yl)aurate(I)] dichloro­methane disolvate

**DOI:** 10.1107/S1600536809045474

**Published:** 2009-11-04

**Authors:** Christoph E. Strasser, Karolien Coetzee, Stephanie Cronje, Helgard G. Raubenheimer

**Affiliations:** a Department of Chemistry and Polymer Science, University of Stellenbosch, Private Bag X1, Matieland 7602, South Africa

## Abstract

The title compound, (C_44_H_38_P_2_)[AuCl(C_6_F_5_)]_2_·2CH_2_Cl_2_, crystallizes with a twofold rotation axis through the central benzene ring in the bis-phospho­nium dication. In the crystal, the dications and anions are ordered into columns running parallel to the *c* axis by contacts of the pro-ylidic CH_2_ groups with the Cl atom of one and an *ortho*-F atom of another anion. The space between the columns is occupied by CH_2_Cl_2_ solvent mol­ecules.

## Related literature

For related structures, see: Briggs *et al.* (1988 ▶); Phillips *et al.* (2008 ▶). For the synthesis of the [AuCl(C_6_F_5_)]^−^ anion, see: Usón *et al.* (1977 ▶). For synthetic details, see: Friedrich & Henning (1959 ▶); Horner *et al.* (1962 ▶); Usón *et al.* (1989 ▶).
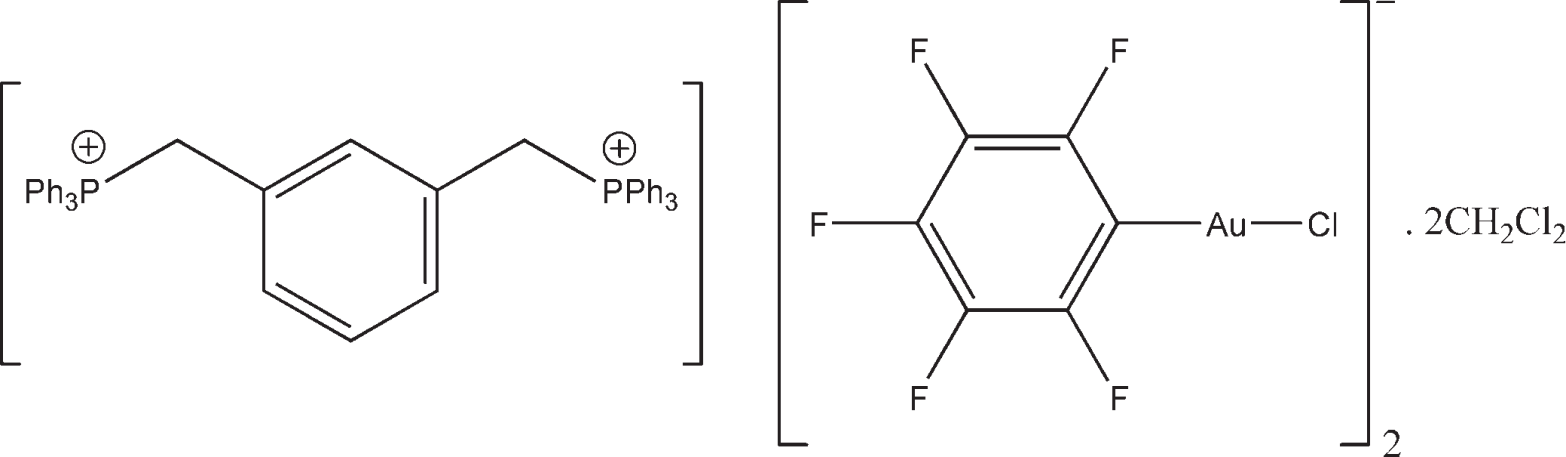


## Experimental

### 

#### Crystal data

(C_44_H_38_P_2_)[AuCl(C_6_F_5_)]_2_·2CH_2_Cl_2_
*M* *_r_* = 1597.5
Orthorhombic, 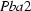

*a* = 14.506 (3) Å
*b* = 22.083 (4) Å
*c* = 8.9439 (18) Å
*V* = 2865.1 (10) Å^3^
*Z* = 2
Mo *K*α radiation
μ = 5.52 mm^−1^
*T* = 203 K
0.35 × 0.12 × 0.12 mm

#### Data collection

Nonius Kappa CCD diffractometer
Absorption correction: multi-scan (*DENZO*; Otwinowski & Minor, 1997 ▶) *T*_min_ = 0.207, *T*_max_ = 0.516
60733 measured reflections
6565 independent reflections
5638 reflections with *I* > 2σ(*I*)
*R*_int_ = 0.042

#### Refinement

*R*[*F* ^2^ > 2σ(*F* ^2^)] = 0.024
*wR*(*F* ^2^) = 0.052
*S* = 1.03
6565 reflections
353 parameters
1 restraint
H-atom parameters constrained
Δρ_max_ = 0.50 e Å^−3^
Δρ_min_ = −0.50 e Å^−3^
Absolute structure: Flack (1983 ▶), 3067 Friedel pairs
Flack parameter: 0.034 (5)

### 

Data collection: *COLLECT* (Nonius, 1998 ▶); cell refinement: *DENZO* (Otwinowski & Minor, 1997 ▶); data reduction: *DENZO*; program(s) used to solve structure: *SHELXS97* (Sheldrick, 2008 ▶); program(s) used to refine structure: *SHELXL97* (Sheldrick, 2008 ▶); molecular graphics: *X-SEED* (Atwood & Barbour, 2003 ▶; Barbour, 2001 ▶); software used to prepare material for publication: *X-SEED*.

## Supplementary Material

Crystal structure: contains datablocks I, global. DOI: 10.1107/S1600536809045474/om2280sup1.cif

Structure factors: contains datablocks I. DOI: 10.1107/S1600536809045474/om2280Isup2.hkl

Additional supplementary materials:  crystallographic information; 3D view; checkCIF report

## Figures and Tables

**Table 1:** Hydrogen-bond geometry (Å, °)

*D*—H⋯*A*	*D*—H	H⋯*A*	*D*⋯*A*	*D*—H⋯*A*
C5—H5*A*⋯Cl1	0.98	2.60	3.579 (5)	174
C5—H5*B*⋯F1^i^	0.98	2.29	3.263 (4)	171

Symmetry code: (i) 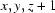.

**Fig. 1.**
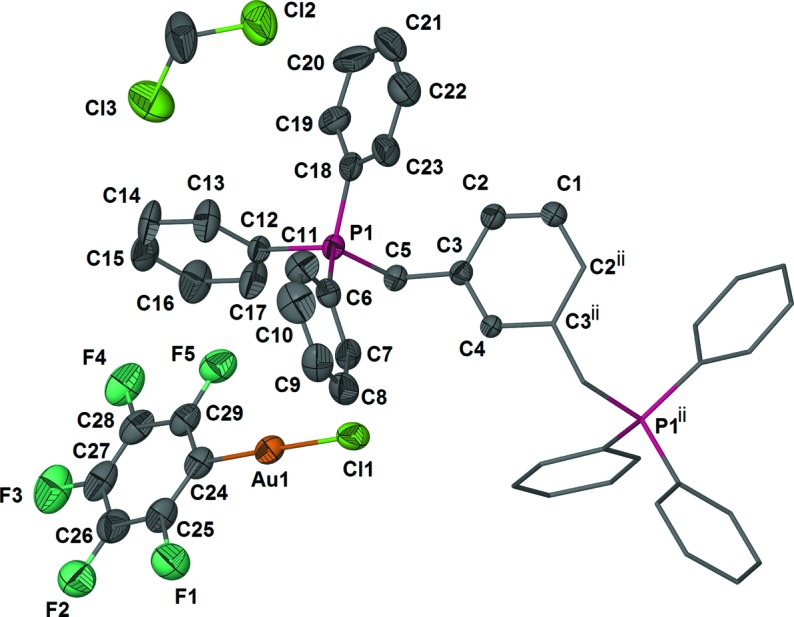
The molecular structure omitting hydrogen atoms and non-covalent interactions. Ellipsoids are drawn at the 50% probability level; the part of the cation related by a twofold rotation is shown in stick representation with symmetry code: ii = -x + 1, -y, z.

**Fig. 2.**
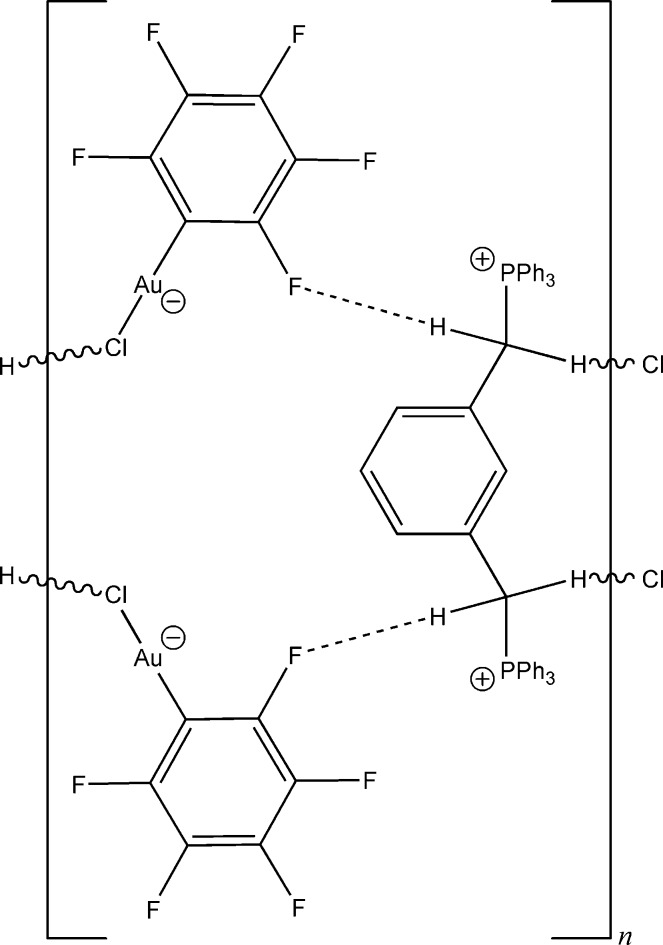
The hydrogen-bonding scheme in (I).

## supplementary crystallographic information

### Comment

Only one half of the bisphosphonium dication in the title compound shown in Scheme 1 is unique, a twofold rotation axis passes through C1 and C4 of the central benzene ring (Figure 1). The pro-ylidic methylene group is engaged in two contacts directed towards the chloro ligand of one [AuCl(C_6_F_5_)]^-^ complex and the other to the *ortho*-fluoro substituent of another anion related by a unit cell translation along the crystallographic *c* axis, Table 1 shows the geometric parameters. The dication thus forms contacts to two anion pairs related by a 2-fold operation. Each pair forms contacts to another dication forming a one-dimensional polar chain illustrated in Scheme 2. The dichloromethane solvent molecules fill channels that run parallel to the anion-cation columns. Cl2 of the CH_2_Cl_2_ molecules is engaged in a Cl···π contact with an electron-withdrawing C_6_F_5_ group [Cl—C_6_ (centroid) distance 3.364 Å)]

A crystal structure that contains the present dication has not been reported. The orientation of the triphenylphosphonium groups in opposite directions is most likely favoured due to steric requirements.

The present crystal structure is one of few that contains the [AuCl(C_6_F_5_)]^-^ anion. One has been reported by Briggs *et al.* (1988) with the (phenylmethyl)triphenylphosphonium cation and another one was reported by Phillips *et al.* (2008) with the tetrabutylammonium cation. The Au—Cl and Au—C bond lengths are comparable to the values found in the present structure, the Au—Cl distance in the structure of Phillips *et al.* is longer at 2.3194 (16) Å. No structure incorporating the [AuCl(C_6_F_5_)]^-^ anion exhibits aurophilic interactions. In the structure of Briggs *et al.*, H···Cl interactions between the pro-ylidic methylene group and the anions also seem to be present, but no hydrogen atom coordinates are supplied for the respective carbon atoms. The [AuCl(C_6_F_5_)]^-^ anion is a valuable starting material for the synthesis of complexes containing the AuC_6_F_5_ fragment (Usón *et al.*, 1977).

### Experimental

The title compound was isolated during an attempted preparation of the bis-ylide complex with Au(C_6_F_5_) fragments. 1,3-Bis[(triphenylphosphonio)methyl]benzene(2+) dibromide was prepared according to a modified literature procedure (Friedrich *et al.*, 1959; Horner *et al.*, 1962). The bromide was exchanged by excess NaBF_4_ in 50% aqueous ethanol. The bis(tetrafluoroborate) was suspended in dry thf and cooled to -45 °C. The mixture turned dark brown upon addition of 1.6 *M n*-butyllithium, was subsequently stirred for 10 min and [Au(C_6_F_5_)(tht)] (tht = tetrahydrothiophene; Usón *et al.*, 1989) in thf was added. The dry residue after removal of solvent was extracted with diethyl ether and dichloromethane. Crystals of the title compound were obtained after two recrystallizations from dichloromethane/hexane.

The chloride in the title compound originates from incomplete reaction during the preparation of [Au(C_6_F_5_)(tht)], in which [AuCl(tht)] is used. Traces of LiCl are thought to have been carried over to form the title compound which crystallizes preferentially. The Au1—Cl1 bond length of 2.3051 (18) Å excludes the presence of bromide from the phosphonium salt.

### Refinement

All H atoms were positioned geometrically (C—H = 0.94, and 0.98 Å for CH and CH_2_ groups, respectively) and constrained to ride on their parent atoms; *U*_iso_(H) values were set at 1.2 times *U*_eq_(C).

### Crystal data

**Table:**

(C_44_H_38_P_2_)[AuCl(C_6_F_5_)]_2_·2CH_2_Cl_2_	*Z* = 2
*M**_r_* = 1597.5	*F*(000) = 1540
Orthorhombic, *P**b**a*2	*D*_x_ = 1.852 Mg m^−^^3^
Hall symbol: P 2 -2ab	Mo *K*α radiation, λ = 0.71073 Å
*a* = 14.506 (3) Å	µ = 5.52 mm^−^^1^
*b* = 22.083 (4) Å	*T* = 203 K
*c* = 8.9439 (18) Å	Needle, colourless
*V* = 2865.1 (10) Å^3^	0.35 × 0.12 × 0.12 mm

### Data collection

**Table:**

Nonius Kappa CCD diffractometer	6565 independent reflections
Radiation source: fine-focus sealed tube	5638 reflections with *I* > 2σ(*I*)
graphite	*R*_int_ = 0.042
φ and ω scans	θ_max_ = 27.5°, θ_min_ = 3.7°
Absorption correction: multi-scan (*DENZO*; Otwinowski & Minor, 1997)	*h* = −18→18
*T*_min_ = 0.207, *T*_max_ = 0.516	*k* = −28→28
60733 measured reflections	*l* = −11→11

### Refinement

**Table:**

Refinement on *F*^2^	Secondary atom site location: difference Fourier map
Least-squares matrix: full	Hydrogen site location: inferred from neighbouring sites
*R*[*F*^2^ > 2σ(*F*^2^)] = 0.024	H-atom parameters constrained
*wR*(*F*^2^) = 0.052	*w* = 1/[σ^2^(*F*_o_^2^) + (0.0174*P*)^2^ + 1.2785*P*] where *P* = (*F*_o_^2^ + 2*F*_c_^2^)/3
*S* = 1.03	(Δ/σ)_max_ = 0.001
6565 reflections	Δρ_max_ = 0.50 e Å^−^^3^
353 parameters	Δρ_min_ = −0.50 e Å^−^^3^
1 restraint	Absolute structure: Flack (1983), 3067 Friedel pairs
Primary atom site location: structure-invariant direct methods	Flack parameter: 0.034 (5)

### Special details

**Table:**

Geometry. All e.s.d.'s (except the e.s.d. in the dihedral angle between two l.s. planes) are estimated using the full covariance matrix. The cell e.s.d.'s are taken into account individually in the estimation of e.s.d.'s in distances, angles and torsion angles; correlations between e.s.d.'s in cell parameters are only used when they are defined by crystal symmetry. An approximate (isotropic) treatment of cell e.s.d.'s is used for estimating e.s.d.'s involving l.s. planes.
Refinement. Refinement of *F*^2^ against ALL reflections. The weighted *R*-factor *wR* and goodness of fit *S* are based on *F*^2^, conventional *R*-factors *R* are based on *F*, with *F* set to zero for negative *F*^2^. The threshold expression of *F*^2^ > 2σ(*F*^2^) is used only for calculating *R*-factors(gt) *etc*. and is not relevant to the choice of reflections for refinement. *R*-factors based on *F*^2^ are statistically about twice as large as those based on *F*, and *R*- factors based on ALL data will be even larger.

### Fractional atomic coordinates and isotropic or equivalent isotropic displacement parameters (Å^2^)

**Table:**

	*x*	*y*	*z*	*U*_iso_*/*U*_eq_	
P1	0.29554 (5)	0.10315 (3)	0.61705 (15)	0.02913 (17)	
C1	0.5000	0.0000	0.9430 (6)	0.0381 (13)	
C2	0.4610 (2)	0.04836 (16)	0.8664 (4)	0.0343 (8)	
C3	0.4600 (2)	0.04836 (15)	0.7119 (4)	0.0277 (7)	
C4	0.5000	0.0000	0.6346 (10)	0.0269 (9)	
C5	0.42041 (17)	0.10111 (12)	0.6264 (6)	0.0302 (6)	
C6	0.2517 (2)	0.03869 (15)	0.5151 (4)	0.0330 (8)	
C7	0.3065 (3)	0.01043 (16)	0.4097 (4)	0.0387 (9)	
C8	0.2733 (3)	−0.0392 (2)	0.3333 (5)	0.0518 (11)	
C9	0.1845 (3)	−0.05876 (18)	0.3566 (5)	0.0536 (11)	
C10	0.1293 (3)	−0.0300 (2)	0.4562 (5)	0.0597 (12)	
C11	0.1620 (3)	0.0191 (2)	0.5372 (5)	0.0497 (10)	
C12	0.2605 (2)	0.17233 (15)	0.5272 (4)	0.0319 (8)	
C13	0.1674 (3)	0.1861 (2)	0.5250 (6)	0.0599 (12)	
C14	0.1374 (3)	0.2384 (2)	0.4552 (6)	0.0643 (13)	
C15	0.2002 (3)	0.2769 (2)	0.3882 (5)	0.0539 (11)	
C16	0.2905 (3)	0.2621 (2)	0.3868 (6)	0.0643 (13)	
C17	0.3224 (3)	0.20981 (19)	0.4564 (5)	0.0536 (12)	
C18	0.2512 (2)	0.10380 (16)	0.8034 (4)	0.0341 (8)	
C19	0.2495 (3)	0.1577 (2)	0.8821 (5)	0.0476 (10)	
C20	0.2253 (3)	0.1578 (3)	1.0317 (6)	0.0675 (14)	
C21	0.2029 (3)	0.1045 (3)	1.1023 (6)	0.0701 (14)	
C22	0.2034 (3)	0.0508 (3)	1.0243 (5)	0.0650 (14)	
C23	0.2284 (3)	0.04949 (19)	0.8757 (5)	0.0466 (10)	
H1	0.5000	0.0000	1.0481	0.046*	
H2	0.4354	0.0810	0.9196	0.041*	
H4	0.5000	0.0000	0.5295	0.032*	
H5A	0.4448	0.1000	0.5242	0.036*	
H5B	0.4420	0.1387	0.6729	0.036*	
H7	0.3661	0.0250	0.3902	0.046*	
H8	0.3115	−0.0597	0.2651	0.062*	
H9	0.1619	−0.0923	0.3031	0.064*	
H10	0.0685	−0.0434	0.4705	0.072*	
H11	0.1237	0.0389	0.6064	0.060*	
H13	0.1247	0.1600	0.5708	0.072*	
H14	0.0742	0.2478	0.4534	0.077*	
H15	0.1801	0.3132	0.3438	0.065*	
H16	0.3326	0.2876	0.3378	0.077*	
H17	0.3856	0.2002	0.4551	0.064*	
H19	0.2648	0.1942	0.8340	0.057*	
H20	0.2241	0.1945	1.0853	0.081*	
H21	0.1871	0.1047	1.2042	0.084*	
H22	0.1866	0.0147	1.0728	0.078*	
H23	0.2302	0.0126	0.8233	0.056*	
Au1	0.488135 (8)	0.198870 (5)	0.12628 (4)	0.04045 (5)	
Cl1	0.50324 (7)	0.10795 (5)	0.24986 (12)	0.0484 (3)	
F1	0.4675 (2)	0.23148 (12)	−0.2198 (3)	0.0672 (7)	
F2	0.45501 (18)	0.33720 (11)	−0.3633 (5)	0.0731 (7)	
F3	0.4605 (2)	0.44303 (12)	−0.2066 (4)	0.0832 (9)	
F4	0.47789 (18)	0.44033 (11)	0.0945 (5)	0.0713 (11)	
F5	0.48874 (17)	0.33557 (15)	0.2410 (4)	0.0608 (8)	
C24	0.4787 (3)	0.27811 (19)	0.0173 (5)	0.0419 (10)	
C25	0.4702 (3)	0.28254 (19)	−0.1338 (6)	0.0474 (10)	
C26	0.4645 (3)	0.3359 (2)	−0.2122 (6)	0.0548 (11)	
C27	0.4675 (3)	0.38949 (19)	−0.1351 (6)	0.0551 (12)	
C28	0.4747 (3)	0.3880 (2)	0.0179 (6)	0.0522 (12)	
C29	0.4806 (3)	0.3329 (2)	0.0903 (5)	0.0452 (14)	
Cl2	0.24384 (10)	0.33560 (10)	1.00079 (18)	0.1041 (6)	
Cl3	0.20261 (13)	0.38659 (9)	0.71284 (19)	0.1085 (6)	
C30	0.1618 (4)	0.3621 (3)	0.8821 (7)	0.103 (2)	
H30B	0.1295	0.3957	0.9308	0.123*	
H30A	0.1166	0.3299	0.8646	0.123*	

### Atomic displacement parameters (Å^2^)

**Table:**

	*U*^11^	*U*^22^	*U*^33^	*U*^12^	*U*^13^	*U*^23^
P1	0.0292 (4)	0.0248 (4)	0.0334 (4)	0.0028 (3)	0.0009 (6)	0.0018 (6)
C1	0.037 (3)	0.051 (4)	0.026 (3)	0.007 (2)	0.000	0.000
C2	0.0310 (18)	0.037 (2)	0.035 (2)	0.0065 (16)	−0.0015 (15)	−0.0067 (16)
C4	0.0248 (19)	0.030 (2)	0.026 (2)	0.0013 (15)	0.000	0.000
C3	0.0250 (17)	0.0260 (18)	0.0322 (19)	0.0016 (14)	−0.0016 (14)	−0.0007 (15)
C5	0.0280 (14)	0.0258 (14)	0.0369 (16)	0.0011 (11)	0.000 (3)	0.000 (3)
C6	0.036 (2)	0.0265 (18)	0.037 (2)	−0.0015 (15)	−0.0052 (16)	0.0040 (15)
C7	0.042 (2)	0.032 (2)	0.042 (2)	0.0031 (16)	−0.0067 (17)	0.0000 (17)
C8	0.060 (3)	0.047 (2)	0.048 (3)	0.011 (2)	−0.015 (2)	−0.010 (2)
C9	0.062 (3)	0.036 (2)	0.062 (3)	−0.004 (2)	−0.021 (2)	−0.008 (2)
C10	0.045 (3)	0.060 (3)	0.074 (3)	−0.025 (2)	−0.007 (2)	0.003 (3)
C11	0.041 (2)	0.050 (2)	0.057 (3)	−0.008 (2)	0.001 (2)	−0.003 (2)
C12	0.035 (2)	0.0254 (18)	0.035 (2)	0.0057 (15)	−0.0021 (16)	0.0020 (15)
C13	0.039 (3)	0.052 (3)	0.088 (3)	0.006 (2)	−0.002 (2)	0.029 (2)
C14	0.043 (3)	0.051 (3)	0.099 (4)	0.019 (2)	−0.009 (2)	0.016 (3)
C15	0.064 (3)	0.037 (2)	0.061 (3)	0.017 (2)	−0.004 (2)	0.009 (2)
C16	0.063 (3)	0.041 (3)	0.089 (4)	0.004 (2)	0.009 (3)	0.028 (2)
C17	0.039 (2)	0.042 (2)	0.080 (3)	0.0096 (18)	0.008 (2)	0.021 (2)
C18	0.0258 (18)	0.039 (2)	0.038 (2)	0.0060 (15)	−0.0003 (14)	0.0046 (17)
C19	0.044 (2)	0.051 (3)	0.048 (3)	0.0028 (19)	0.005 (2)	−0.009 (2)
C20	0.056 (3)	0.093 (4)	0.054 (3)	0.014 (3)	0.000 (2)	−0.034 (3)
C21	0.055 (3)	0.118 (4)	0.037 (3)	0.016 (3)	0.004 (2)	0.007 (3)
C22	0.056 (3)	0.090 (4)	0.049 (3)	0.005 (3)	0.002 (2)	0.026 (3)
C23	0.048 (2)	0.046 (3)	0.046 (3)	0.0036 (19)	0.0011 (19)	0.0130 (19)
Au1	0.03975 (8)	0.03337 (8)	0.04823 (9)	0.00044 (5)	0.0041 (2)	−0.00390 (14)
Cl1	0.0664 (7)	0.0379 (6)	0.0408 (6)	0.0042 (4)	0.0087 (5)	0.0024 (4)
F1	0.090 (2)	0.0504 (17)	0.0612 (17)	0.0021 (14)	−0.0002 (14)	−0.0054 (14)
F2	0.0798 (16)	0.0718 (15)	0.0676 (18)	0.0066 (13)	0.011 (2)	0.012 (2)
F3	0.085 (2)	0.0458 (16)	0.119 (3)	0.0025 (15)	0.0096 (19)	0.0261 (16)
F4	0.0790 (17)	0.0310 (12)	0.104 (3)	−0.0003 (10)	0.0047 (17)	−0.0101 (16)
F5	0.0653 (18)	0.0474 (19)	0.070 (2)	0.0042 (12)	−0.0004 (14)	−0.0151 (18)
C24	0.033 (2)	0.036 (2)	0.058 (3)	−0.0002 (16)	0.0033 (18)	0.000 (2)
C25	0.044 (2)	0.030 (2)	0.068 (3)	−0.0009 (18)	0.004 (2)	−0.006 (2)
C26	0.044 (3)	0.057 (3)	0.063 (3)	0.004 (2)	0.008 (2)	0.005 (2)
C27	0.040 (2)	0.033 (2)	0.093 (4)	−0.0023 (18)	0.007 (2)	0.008 (2)
C28	0.039 (2)	0.037 (3)	0.081 (4)	−0.0027 (18)	0.006 (2)	−0.006 (2)
C29	0.033 (2)	0.038 (2)	0.065 (4)	−0.0004 (15)	0.0047 (19)	−0.005 (2)
Cl2	0.0567 (9)	0.1708 (17)	0.0849 (11)	0.0179 (10)	−0.0005 (8)	0.0315 (11)
Cl3	0.1261 (14)	0.1259 (15)	0.0733 (9)	0.0085 (11)	0.0209 (9)	0.0275 (9)
C30	0.047 (3)	0.155 (6)	0.106 (5)	0.015 (4)	0.009 (3)	0.066 (4)

### Geometric parameters (Å, °)

**Table:**

P1—C5	1.814 (3)	C7—H7	0.9400
P1—C6	1.806 (3)	C8—H8	0.9400
P1—C12	1.799 (3)	C9—H9	0.9400
P1—C18	1.787 (4)	C10—H10	0.9400
C1—C2	1.389 (4)	C11—H11	0.9400
C1—C2^i^	1.389 (4)	C13—H13	0.9400
C3—C2	1.382 (5)	C14—H14	0.9400
C3—C4	1.399 (5)	C15—H15	0.9400
C3—C5	1.507 (5)	C16—H16	0.9400
C4—C3^i^	1.399 (5)	C17—H17	0.9400
C6—C7	1.382 (5)	C19—H19	0.9400
C6—C11	1.385 (5)	C20—H20	0.9400
C7—C8	1.378 (6)	C21—H21	0.9400
C8—C9	1.375 (6)	C22—H22	0.9400
C9—C10	1.356 (6)	C23—H23	0.9400
C10—C11	1.387 (6)	Au1—Cl1	2.3024 (12)
C12—C13	1.385 (6)	Au1—C24	2.008 (4)
C12—C17	1.376 (5)	F1—C25	1.365 (5)
C13—C14	1.382 (6)	F2—C26	1.359 (6)
C14—C15	1.383 (6)	F3—C27	1.348 (5)
C15—C16	1.351 (6)	F4—C28	1.343 (5)
C16—C17	1.391 (6)	F5—C29	1.354 (6)
C18—C19	1.383 (5)	C24—C25	1.361 (6)
C18—C23	1.402 (5)	C24—C29	1.375 (7)
C19—C20	1.384 (6)	C25—C26	1.373 (7)
C20—C21	1.376 (7)	C26—C27	1.371 (7)
C21—C22	1.375 (7)	C27—C28	1.373 (7)
C22—C23	1.378 (6)	C28—C29	1.382 (7)
C1—H1	0.9400	Cl2—C30	1.698 (5)
C2—H2	0.9400	Cl3—C30	1.714 (6)
C4—H4	0.9400	C30—H30B	0.9800
C5—H5A	0.9800	C30—H30A	0.9800
C5—H5B	0.9800		
			
P1—C5—H5A	108.5	C9—C8—H8	120.0
P1—C5—H5B	108.5	C9—C10—H10	119.8
C6—P1—C5	110.82 (16)	C10—C9—H9	119.8
C12—P1—C5	108.89 (16)	C10—C11—H11	120.3
C12—P1—C6	110.12 (17)	C11—C10—H10	119.8
C18—P1—C5	108.5 (2)	C12—C13—H13	120.1
C18—P1—C6	110.52 (17)	C12—C17—H17	120.4
C18—P1—C12	107.94 (17)	C13—C14—H14	119.9
C3—C5—P1	115.0 (2)	C14—C13—H13	120.1
C7—C6—P1	119.8 (3)	C14—C15—H15	120.2
C11—C6—P1	120.3 (3)	C15—C14—H14	119.9
C13—C12—P1	117.9 (3)	C15—C16—H16	119.3
C17—C12—P1	122.1 (3)	C16—C15—H15	120.2
C19—C18—P1	119.2 (3)	C16—C17—H17	120.4
C23—C18—P1	120.5 (3)	C17—C16—H16	119.3
C2^i^—C1—C2	120.9 (5)	C18—C19—H19	120.1
C2—C3—C4	119.3 (4)	C18—C23—H23	120.4
C2—C3—C5	120.8 (3)	C19—C20—H20	119.9
C3—C2—C1	119.8 (4)	C20—C19—H19	120.1
C3^i^—C4—C3	120.7 (7)	C20—C21—H21	119.9
C4—C3—C5	119.8 (4)	C21—C20—H20	119.9
C6—C11—C10	119.4 (4)	C21—C22—H22	119.7
C7—C6—C11	119.8 (3)	C22—C21—H21	119.9
C8—C7—C6	119.8 (4)	C22—C23—H23	120.4
C9—C8—C7	120.1 (4)	C23—C22—H22	119.7
C9—C10—C11	120.5 (4)	H5A—C5—H5B	107.5
C10—C9—C8	120.4 (4)	F1—C25—C24	120.2 (4)
C12—C17—C16	119.2 (4)	F1—C25—C26	114.8 (4)
C13—C14—C15	120.2 (4)	F2—C26—C25	122.2 (4)
C14—C13—C12	119.8 (4)	F2—C26—C27	119.0 (4)
C15—C16—C17	121.3 (4)	F3—C27—C26	121.1 (5)
C16—C15—C14	119.6 (4)	F3—C27—C28	119.9 (4)
C17—C12—C13	119.9 (3)	F4—C28—C27	119.4 (4)
C19—C18—C23	119.9 (3)	F4—C28—C29	121.1 (5)
C20—C19—C18	119.9 (4)	F5—C29—C24	120.9 (5)
C21—C20—C19	120.1 (5)	F5—C29—C28	115.7 (5)
C22—C21—C20	120.3 (5)	C24—Au1—Cl1	178.43 (11)
C22—C23—C18	119.2 (4)	C25—C24—Au1	123.4 (3)
C23—C22—C21	120.6 (4)	C29—C24—Au1	122.3 (4)
C1—C2—H2	120.1	C24—C25—C26	125.0 (4)
C2—C1—H1	119.5	C24—C29—C28	123.5 (5)
C2^i^—C1—H1	119.5	C25—C24—C29	114.2 (4)
C3—C2—H2	120.1	C26—C27—C28	118.9 (4)
C3—C4—H4	119.7	C27—C26—C25	118.8 (5)
C3^i^—C4—H4	119.7	C27—C28—C29	119.5 (4)
C3—C5—H5A	108.5	Cl2—C30—Cl3	114.8 (3)
C3—C5—H5B	108.5	Cl2—C30—H30A	108.6
C6—C7—H7	120.1	Cl2—C30—H30B	108.6
C6—C11—H11	120.3	Cl3—C30—H30A	108.6
C7—C8—H8	120.0	Cl3—C30—H30B	108.6
C8—C7—H7	120.1	H30B—C30—H30A	107.6
C8—C9—H9	119.8		
			
P1—C6—C7—C8	179.2 (3)	C9—C10—C11—C6	0.2 (7)
P1—C6—C11—C10	179.1 (3)	C11—C6—C7—C8	−3.6 (6)
P1—C12—C13—C14	−179.1 (4)	C12—C13—C14—C15	−0.2 (7)
P1—C12—C17—C16	178.8 (4)	C13—C12—C17—C16	1.7 (7)
P1—C18—C19—C20	−172.7 (3)	C13—C14—C15—C16	2.3 (7)
P1—C18—C23—C22	173.4 (3)	C14—C15—C16—C17	−2.4 (8)
C5—P1—C6—C7	−25.6 (3)	C15—C16—C17—C12	0.4 (8)
C5—P1—C6—C11	157.3 (3)	C17—C12—C13—C14	−1.8 (7)
C5—P1—C12—C13	−172.4 (3)	C18—C19—C20—C21	0.0 (7)
C5—P1—C12—C17	10.4 (4)	C19—C18—C23—C22	0.8 (6)
C5—P1—C18—C19	80.3 (3)	C19—C20—C21—C22	−0.7 (7)
C5—P1—C18—C23	−92.2 (3)	C20—C21—C22—C23	1.5 (7)
C6—P1—C5—C3	−64.2 (4)	C21—C22—C23—C18	−1.6 (7)
C6—P1—C12—C13	65.9 (4)	C23—C18—C19—C20	0.0 (6)
C6—P1—C12—C17	−111.3 (4)	Au1—C24—C25—F1	0.1 (6)
C6—P1—C18—C19	−158.0 (3)	Au1—C24—C29—F5	−0.2 (5)
C6—P1—C18—C23	29.4 (4)	Au1—C24—C25—C26	−179.3 (3)
C12—P1—C5—C3	174.5 (3)	Au1—C24—C29—C28	179.6 (3)
C12—P1—C6—C7	94.9 (3)	F1—C25—C26—F2	1.3 (6)
C12—P1—C6—C11	−82.2 (3)	F2—C26—C27—F3	0.5 (6)
C12—P1—C18—C19	−37.5 (3)	F3—C27—C28—F4	−2.2 (6)
C12—P1—C18—C23	149.9 (3)	F4—C28—C29—F5	0.5 (6)
C18—P1—C5—C3	57.3 (3)	F1—C25—C26—C27	−179.4 (4)
C18—P1—C6—C7	−145.9 (3)	F2—C26—C27—C28	178.4 (4)
C18—P1—C6—C11	37.0 (4)	F3—C27—C28—C29	179.1 (4)
C18—P1—C12—C13	−54.8 (4)	F4—C28—C29—C24	−179.3 (4)
C18—P1—C12—C17	128.0 (4)	C24—C25—C26—F2	−179.2 (4)
C2—C3—C5—P1	−78.2 (4)	C25—C24—C29—F5	180.0 (4)
C4—C3—C5—P1	104.8 (3)	C25—C26—C27—F3	−178.8 (4)
C2^i^—C1—C2—C3	0.7 (2)	C26—C27—C28—F4	179.9 (4)
C2—C3—C4—C3^i^	0.6 (2)	C27—C28—C29—F5	179.2 (4)
C4—C3—C2—C1	−1.3 (5)	C29—C24—C25—F1	179.9 (3)
C5—C3—C2—C1	−178.3 (3)	C24—C25—C26—C27	0.1 (7)
C5—C3—C4—C3^i^	177.7 (3)	C25—C24—C29—C28	−0.2 (6)
C6—C7—C8—C9	3.1 (6)	C25—C26—C27—C28	−1.0 (7)
C7—C6—C11—C10	2.0 (6)	C26—C27—C28—C29	1.2 (6)
C7—C8—C9—C10	−0.9 (6)	C27—C28—C29—C24	−0.6 (6)
C8—C9—C10—C11	−0.7 (7)	C29—C24—C25—C26	0.5 (6)

Symmetry codes: (i) −*x*+1, −*y*, *z*.

### Hydrogen-bond geometry (Å, °)

**Table:**

*D*—H···*A*	*D*—H	H···*A*	*D*···*A*	*D*—H···*A*
C5—H5A···Cl1	0.98	2.60	3.579 (5)	174
C5—H5B···F1^ii^	0.98	2.29	3.263 (4)	171

Symmetry codes: (ii) *x*, *y*, *z*+1.

## References

[bb1] Atwood, J. L. & Barbour, L. J. (2003). *Cryst. Growth Des.* **3**, 3–8.

[bb2] Barbour, L. J. (2001). *J. Supramol. Chem.* **1**, 189–191.

[bb3] Briggs, D. A., Raptis, R. G. & Fackler, J. P. Jnr (1988). *Acta Cryst.* C**44**, 1313–1315.

[bb4] Flack, H. D. (1983). *Acta Cryst.* A**39**, 876–881.

[bb5] Friedrich, K. & Henning, H. (1959). *Chem. Ber.* **92**, 2756–2760.

[bb6] Horner, L., Hoffmann, H., Klink, W., Ertel, H. & Toscano, V. G. (1962). *Chem. Ber.* **95**, 581–601.

[bb7] Nonius (1998). *COLLECT*, Nonius BV, Delft, The Netherlands.

[bb8] Otwinowski, Z. & Minor, W. (1997). *Methods in Enzymology*, Vol. 276, *Macromolecular Crystallography*, Part A, edited by C. W. Carter Jr & R. M. Sweet, pp. 307–326. New York: Academic Press.

[bb9] Phillips, V., Doerrer, L. H. & Rheingold, A. L. (2008). Private communication (Refcode: PONGAB). CCDC, Cambridge, England.

[bb10] Sheldrick, G. M. (2008). *Acta Cryst.* A**64**, 112–122. 10.1107/S0108767307043930 18156677

[bb11] Usón, R., Laguna, A. & Laguna, M. (1989). *Inorg. Synth.* **26**, 85–91.

[bb12] Usón, R., Laguna, A. & Vicente, J. (1977). *J. Organomet. Chem.* **131**, 471–475.

